# Single-cell transcriptomics reveals a low CD8^+^ T cell infiltrating state mediated by fibroblasts in recurrent renal cell carcinoma

**DOI:** 10.1136/jitc-2021-004206

**Published:** 2022-02-04

**Authors:** Yu-Lu Peng, Long-Bin Xiong, Zhao-Hui Zhou, Kang Ning, Zhen Li, Ze-Shen Wu, Min-Hua Deng, Wen-Su Wei, Ning Wang, Xiang-Peng Zou, Zhi-Song He, Ji-Wei Huang, Jun-Hang Luo, Jian-Ye Liu, Nan Jia, Yun Cao, Hui Han, Sheng-Jie Guo, Pei Dong, Chun-Ping Yu, Fang-Jian Zhou, Zhi-Ling Zhang

**Affiliations:** 1 Department of Urology, Sun Yat-sen University Cancer Center, Guangzhou, Guangdong, China; 2 State Key Laboratory of Oncology in South China, Collaborative Innovation Center for Cancer Medicine, Sun Yat-Sen University Cancer Center, Guangzhou, China; 3 Department of Urology, Huazhong University of Science and Technology Union Shenzhen Hospital, Shenzhen, Guangdong, China; 4 Department of Urology, Peking University First Hospital, Beijing, China; 5 Department of Urology, Shanghai Jiao Tong University School of Medicine Affiliated Renji Hospital, Shanghai, China; 6 Department of Urology, Sun Yat-sen University First Affiliated Hospital, Guangzhou, Guangdong, China; 7 Department of Urology, Central South University Third Xiangya Hospital, Changsha, Hunan, China; 8 Department of Nephrology, Southern Medical University Nanfang Hospital, Guangzhou, Guangdong, China; 9 Department of Pathology, Sun Yat-sen University Cancer Center, Guangzhou, Guangdong, China

**Keywords:** kidney neoplasms, tumor microenvironment, immunotherapy, translational medical research

## Abstract

**Purpose:**

Recurrent renal cell carcinoma(reRCC) is associated with poor prognosis and the underlying mechanism is not yet clear. A comprehensive understanding of tumor microenvironment (TME) of reRCC may aid in designing effective anticancer therapies, including immunotherapies. Single-cell transcriptomics holds great promise for investigating the TME, however, this technique has not been used in reRCC. Here, we aimed to explore the difference in the TME and gene expression pattern between primary RCC (pRCC) and reRCC at single-cell level.

**Experimental design:**

We performed single-cell RNA sequencing analyses of 32,073 cells from 2 pRCC, 2 reRCC, and 3 adjacent normal kidney samples. 41 pairs of pRCC and reRCC samples were collected as a validation cohort to assess differences observed in single-cell sequencing. The prognostic significance of related cells and markers were studied in 47 RCC patients underwent immunotherapy. The function of related cells and markers were validated via in vitro and in vivo experiments.

**Results:**

reRCC had reduced CD8^+^ T cells but increased cancer-associated fibroblasts (CAFs) infiltration compared with pRCC. Reduced CD8^+^ T cells and increased CAFs infiltration were significantly associated with a worse response from immunotherapy. Remarkably, CAFs showed substantial expression of LGALS1 (Gal1). In vitro, CAFs could induce CD8^+^ T cells apoptosis via Gal1. In vivo, knockdown of Gal1 in CAFs suppressed tumor growth, increased CD8^+^ T cells infiltration, reduced the proportion of apoptotic CD8^+^ T cells and enhanced the efficacy of immunotherapy.

**Conclusions:**

We delineated the heterogeneity of reRCC and highlighted an innovative mechanism that CAFs acted as a suppressor of CD8^+^ T cells via Gal1. Targeting Gal1 combined with anti-PD1 showed promising efficacy in treating RCC.

## Introduction

Renal cell carcinoma (RCC) is one of the most common malignant tumors of the urinary system, with an annual worldwide increase of ~2% in incidence during the last two decades.^1^ So far, surgery remains the most effective therapy for clinically localized RCC.^2^ However, about 20% of patients with RCC develop recurrence after surgical excision.^3–6^ Recurrent RCC (reRCC) represents a major clinical challenge and patients who experienced failure from local therapy often have poor prognosis.^5^ Although immune checkpoint blockade therapy and combination regimens have increased the survival of RCC patients, the prognosis of patients with advanced RCC, including reRCC, remains poor.^7–9^ A thorough investigation of reRCC could enhance our understanding on the mechanisms of tumor development and progression, and more importantly, facilitate the discovery of more effective therapeutic regimens for RCC.

The tumor microenvironment (TME) is a complex ecosystem composed of several cell types, including immune cells, cancer-associated fibroblasts (CAFs), endothelial cells, and extracellular matrix (ECM), which plays a critical role in cancer progression.^10^ Among the immune cells infiltrating the TME, T cell, especially CD8^+^ T cell, is a key antitumor immune component. The success of immunotherapy depends on the activation of a potent cytotoxic T cell response.^11^ CAFs are the most abundant stromal cells in the TME and critically contribute to cancer progression.^12^ Numerous studies have shown that CAFs can promote cancer via multiple processes including, but not limited to, cancer stem-cell renewal,^13^ chemoresistance,^14^ and immune-cell evasion.^14 15^ Furthermore, there have been series of studies suggesting that CAFs can blunt immunotherapy efficacy, and thus, CAFs have been regarded as an emerging target of anti-cancer immunotherapy.^14 16^ Although various mechanisms related to the immunosuppressive features of CAFs have been reported, the mechanism of immunotherapy resistance mediated by CAFs is complicated and deserves further investigation.

Tumors secrete various growth factors and cytokines to shape an immunosuppressive TME. Galectin-1 (Gal1), a well-known immunosuppressor, can induce apoptosis of activated T cells and suppress T cell-mediated cytotoxic immune responses.^17–19^ It is reported that Gal1 was highly expressed in multiple cell types including CAFs and could be secreted into the surrounding milieu of tumor.^20–23^ Gal1 secreted by CAFs could promote epithelial-mesenchymal transformation in gastric cancer^24^ and targeting Gal1 in CAFs has been shown to be able to inhibit oral squamous cell carcinoma metastasis.^25^ However, the role of CAFs-derived Gal1 in the tumor immune microenvironment remains undetermined.

Single-cell RNA sequencing (scRNA-seq) is a promising method to investigate the cellular components and their interactions in the TME with high resolution.^26 27^ ScRNA-seq has been used to distinguish the precise cellular identities and compositions of human kidney tumors.^28^ Kim *et al*^29^ unraveled the utility and validity of single-cell sequencing for the design of personalized therapeutic strategies in patients with metastatic RCC. A series of recent studies have examined the characteristics of TME in RCC patients using single-cell sequencing,^30–33^ however, all these studies have focused on primary or metastatic tumors. Moreover, previous studies comparing primary versus recurrent tumors suggested that secondary tumors can be completely different in TME and gene expression from the primary tumor.^26 34^ Thus, a comprehensive depiction of the differences between primary RCC (pRCC) and reRCC at the single-cell resolution level remains in urgent need.

In this study, we used scRNA-seq to profile single cells from pRCC and reRCC. Clinical paired pRCC and reRCC tumor samples were used to validate the differences observed in single-cell sequencing via immunohistochemistry (IHC). The prognostic significance of related cells and markers were studied in RCC patients who received immunotherapy. The function of related cells and markers were assessed via in vivo experiment. This study could improve our understanding on the mechanisms associated with tumor development and recurrence in RCC at the single-cell level.

## Methods

This study was performed in accordance with the ethical standards of the Helsinki Declaration and the ethical guidelines for Medical and Health Research Involving Human Subjects. Written informed consent was obtained for each of the participant patients. All cases were deidentified and personal identifiable details were removed from their case descriptions to ensure anonymity. This study was approved and reviewed by the Ethics Review Board of Sun Yat-sen University Cancer Center (SYSUCC). Mouse experiments were performed in a specific pathogen-free environment at the animal laboratory of the SYSUCC according to institutional guidelines, and all animal experimental protocols were approved and reviewed by the Ethics Review Committee for Animal Experimentation of SYSUCC. All experiments were performed in accordance with the guidelines and regulations indicated by these committees.

### Experimental design

**Figure 1:**
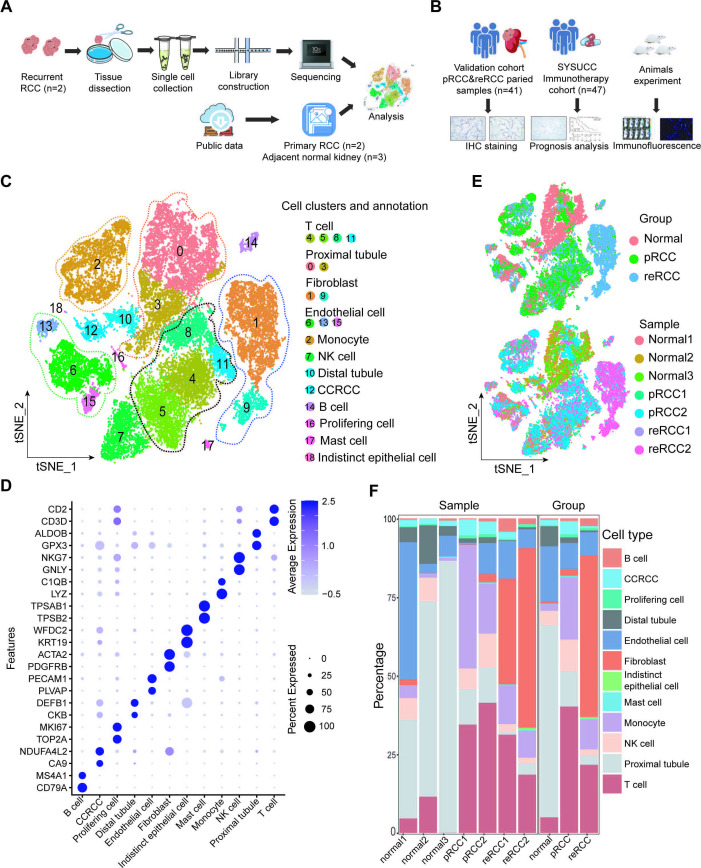
scRNA-seq profiling of the tumor ecosystem in adjacent normal kidneys, pRCC and reRCC. (A, B) Schematic representation of the experimental design. (C) The t-SNE plot, showing the annotation and color codes for cell types in the RCC ecosystem. (D) Dotplot showing the expression of marker genes in the indicated cell types. (E) The t-SNE plot, showing cell origins by color, groups origin and patients origin. (F) Histogram showing the percentage of cell types in samples and groups. IHC, immunohistochemistry; pRCC, primary renal cell carcinoma; RCC, renal cell carcinoma; reRCC, recurrent renal cell carcinoma; SYSUCC, Sun Yat-sen University Cancer Center; t-SNE: T-distributed stochastic neighbor embedding.

We performed single-cell sequencing on tumor tissue of two patients, who had local reRCC after nephrectomy and had recurrent tumor resection at SYSUCC. In parallel, the scRNA-seq data of 2 pRCC and 3 adjacent normal kidney samples described by Young *et al*^28^ which is the first study characterizing treatment naïve pRCC using scRNA-seq, were downloaded for integrative analyses (figure 1A, online supplemental table S1). The validation cohort containing 41 pairs of pRCC and reRCC samples were retrospectively collected to assess differences observed in single-cell sequencing via IHC. The prognostic significance of related cells and markers were studied in SYSUCC immunotherapy cohort including 47 advanced RCC patients who underwent anti-PD1 immunotherapy and in the The Cancer Genome Atlas (TCGA) cohort. The function of related cells and markers were validated via in vitro and in vivo experiments. For further details regarding the materials and methods, please refer to the online supplemental methods.

10.1136/jitc-2021-004206.supp1 Supplementary data

10.1136/jitc-2021-004206.supp2 Supplementary data

## Results

### Patient cohorts

The study was designed and conducted as illustrated in figure 1A, B. reRCC tissues were obtained from two patients diagnosed with local relapsed clear cell RCC in SYSUCC. The pathological type was renal clear cell carcinoma. Moreover, we recruited 41 patients with paired pRCC and reRCC samples to perform IHC to confirm differences found in the scRNA-seq. In this study, recurrent cancer within the renal fossa qualify as reRCC after partial nephrectomy or radical nephrectomy. In these 41 patients, 36 patients (87.8%) had a reRCC within 5 years. According to pathological typing, 34 patients (82.9%) had clear cell carcinoma, and 7 (17.1%) a papillary RCC. All patients had no change in the pathological type when experience tumor recurrence. Forty-seven RCC patients who received immunotherapy in our center were included in the SYSUCC immunotherapy cohort. We defined progression-free survival (PFS) as the time from immunotherapy initiation to disease progression or death from any cause. The available clinical features of the SYSUCC immunotherapy cohort are summarized in online supplemental table S2. scRNA-seq profiling of the tumor ecosystem in pRCC and reRCC.

After quality control and removal of the batch effect between samples, 32,073 single cells were clustered into 19 major clusters using the T-distributed stochastic neighbor embedding (t-SNE) method. Cluster-specific genes were used to annotate cell types with classic markers described in previous studies.^26 31 35–37^ We identified four types of epithelial cells in five clusters, including proximal tubule cells (ALDOB^+^ and GPX3^+^), cancer cells (CA9^+^ and NDUFA4L2^+^), distal tubule cells (DEFB1^+^ and CKB^+^), and indistinct epithelial cells (KRT19^+^ and WFDC2^+^); 5 types of immune cells in eight clusters, including T cells (CD3D^+^ and CD2^+^), NK cell (NKG7^+^ and GNLY^+^), monocytes (LYZ^+^ and C1QB^+^), B cells (CD79A^+^ and MS4A1^+^), and mast cells (TPSAB1^+^ and TPSB2^+^); fibroblasts (PDGFRB^+^ and ACTA2^+^); vascular endothelial (PECAM1^+^ and PLVAP^+^) and profiling cells (TOP2A^+^ and MKI67^+^) (figure 1C, D, online supplemental table S4). In adjacent normal kidney samples, proximal tubule cells and distal tubule cells were the most abundant cell types, with a low abundance of immune cell infiltration. In tumor samples, T cells and monocytes were the main infiltrating immune cells. T cells accounted for 21.65% in reRCC and 40.16% in pRCC, while monocytes accounted for 9.53% in reRCC and 19.81% in pRCC. The proportion of vascular endothelial cells in reRCC and pRCC was 7.46% and 8.28%, respectively, indicating the rationality of antiangiogenic therapy in RCC. Remarkably, compared with normal and pRCC samples, reRCC samples harbored a relatively higher proportion of fibroblasts (51.29% vs 2.19%). The infiltration levels of B cells, natural killer (NK) cells, and mast cells were relatively low in all patients.

In summary, all the described cell types were shared across patients, however, the proportion of cell types varied among pRCC and reRCC patients, revealing substantial heterogeneity of their TME compositions (figure 1E, F).

### Identification and characterization of malignant cells in PRCC and reRCC

**Figure 2:**
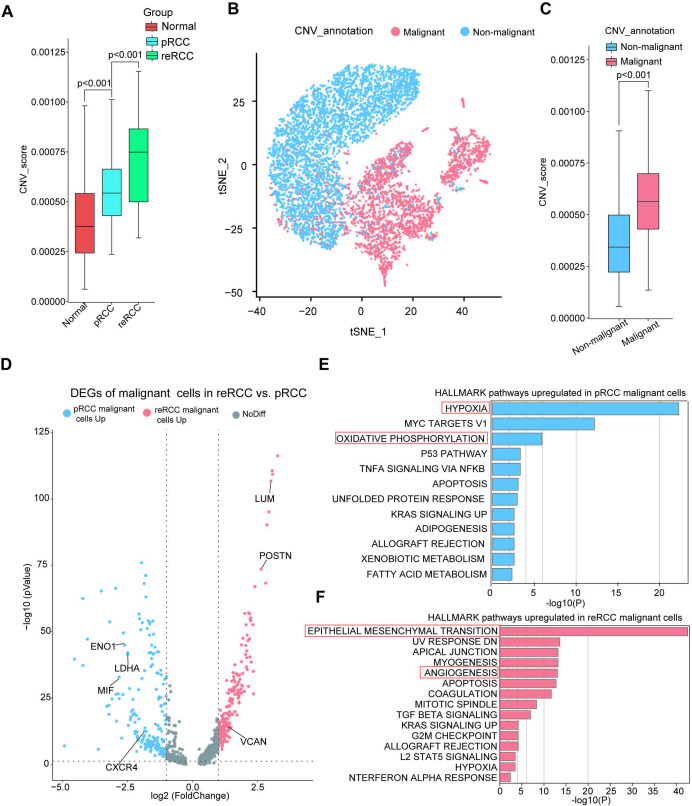
Identification and characterization of malignant cells in pRCC and reRCC. (A) CNV score of epithelial cells in normal, pRCC and reRCC tissues. (B) The t-SNE plot, showing the malignant and non-malignant epithelial cells. (C) CNV score of malignant and non-malignant epithelial cells. (D) The volcano plot shows DEGs between pRCC (blue dots) and reRCC malignant cells (red dots). (E, F). Bar chart showing the enrichment of specific pathways, based on the HALLMARK gene set of upregulated genes of malignant cells in reRCC (E) and pRCC. CNV, copy number variation; DEGs, differentially expressed genes; pRCC, primary renal cell carcinoma; reRCC, recurrent renal cell carcinoma; t-SNE: T-distributed stochastic neighbor embedding.

In this study, we identified four main subtypes of epithelial cells in adjacent normal kidneys, pRCC, and reRCC tissues. Thereafter, copy number variation (CNV) analysis was performed to distinguish between malignant and non-malignant epithelial cells (online supplemental figure S1A). CNVs accumulated in most tumor-derived epithelial cells and showed high heterogeneity among clusters. Our data also indicated that the average CNV level of each epithelial cell in tumor samples was significantly higher than that in normal samples (figure 2A). We further employed K-Means clustering in all epithelial cells to distinguish between malignant and non-malignant epithelial cells based on CNV levels. K-Means clustering revealed seven distinct classes of expression patterns. The results showed a lower CNV score in class 3 and class 5, which were mainly composed of epithelial cells derived from normal samples. When class 3 and class 5 were split by groups, an extremely lower CNV score was found in the normal groups (online supplemental figure S1B, C). Therefore, we defined epithelial cells derived from the adjacent normal kidney in class 3 and 5 as non-malignant epithelial cells, and the rest as malignant cells. A significantly higher CNV score was characterized in malignant cells (figure 2B, C).

10.1136/jitc-2021-004206.supp3 Supplementary data

We further examined the differentially expressed genes (DEGs) in malignant cells between pRCC and reRCC samples (online supplemental table S5). We found that the expression of CXCR4 was significantly higher in malignant cells in pRCC patients (figure 2D). CXCR4 is considered as an oncogene that promotes tumor progression and has been reported as a new therapeutic target for RCC.^38^ MIF, LDHA, and ENO1 were also found upregulated in pRCC (figure 2D). HALLMARK pathways analysis of upregulated expression genes enriched in oxidative phosphorylation and hypoxia suggested a hypoxic characteristic in pRCC (figure 2E). The genes upregulated in malignant cells in reRCC included VCAN, LUM, and POSTN (figure 2D). Pathway enrichment analysis based on HALLMARK gene sets indicated a highly activated state of epithelial-mesenchymal transition and angiogenesis pathways in reRCC patients (figure 2F).

These results showed the heterogeneity in expression pattern and difference in activated pathways between reRCC and pRCC in malignant cells.

### CD8^+^T cells had a lower infiltrating level and were enriched in the apoptosis pathway in reRCC

**Figure 3:**
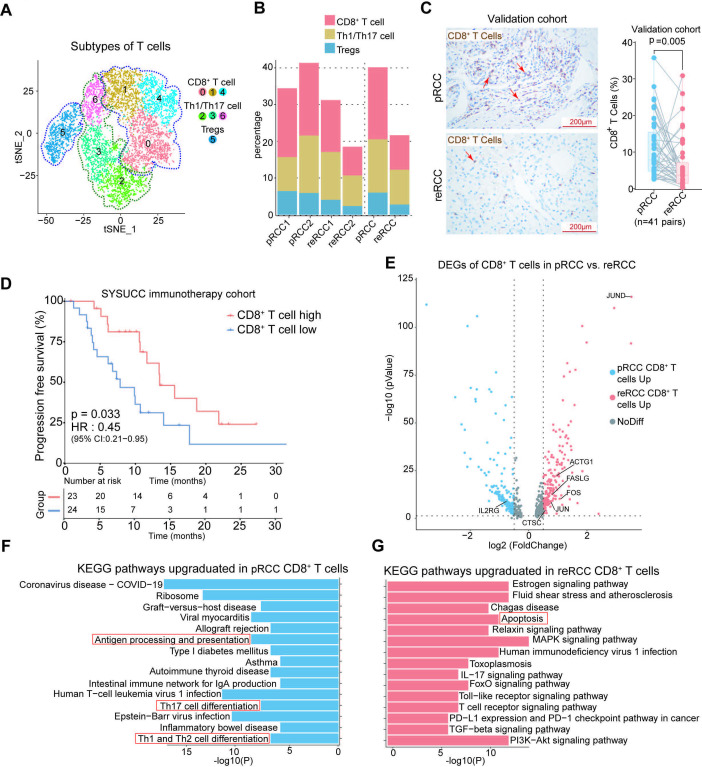
Characteristics of infiltrating T cell in pRCC and reRCC. (A) The t-SNE plot, showing subclustered T cells, is labeled in different colors. Cell type annotations are provided in the figure. (B) Histogram showing the percentage of T cell subtypes in samples and groups. (C) IHC staining of CD8A antibodies, showing the infiltration of CD8^+^ T cells, in paired pRCC and reRCC samples from the validation cohort. Scale bars, 50 µm. (D) PFS curves according to CD8^+^ T cells infiltration in tumor tissues in SYSUCC immunotherapy cohort. CD8 high represented a higher CD8^+^ T cells infiltration and showed a significantly better prognosis. (E) The volcano plot shows differentially expressed genes between pRCC (blue dots) and reRCC CD8^+^ T cells (red dots). (F, G) Bar chart showing the enrichment of specific pathways, based on the KEGG gene set of upregulated genes, in pRCC and reRCC CD8^+^ T cells. IHC, immunohistochemistry; PFS, progression-free survival; pRCC, primary renal cell carcinoma; RCC, renal cell carcinoma; reRCC, recurrent renal cell carcinoma; SYSUCC, Sun Yat-sen University Cancer Center; t-SNE, T-distributed stochastic neighbor embedding.

The re-clustering of T cells revealed seven clusters, including 1 cluster of Tregs (CD4^+^, FOXP3^+^), three clusters of Th1/Th17 cells (CD8^-^, IL7R^+^), and three clusters of CD8^+^ T cells (CD8A^+^ IL7R^+^, CD8A^+^ GZMH^+^ IL7R^-^, and CD8A^+^ HAVCR2^+^) (figure 3A and online supplemental figure S2A). These subtypes were present in all pRCC and reRCC samples (figure 3B). Tregs presented in tumors and displayed increased expression of CD4, FOXP3, and LAIR2 (figure 3B, and online supplemental figure S2B). All 3 subtypes of CD8^+^ T cells showed increased expression of cytotoxic genes GZMK, GZMA, and NKG7 (online supplemental figure S2C). Cluster 0 of CD8^+^ T cells (CD8A^+^ IL7R^+^ cells) was characterized by specifically expressing IL7R and was previously reported to be precursors of memory CD8^+^ T cell.^39^ Cluster 1 of CD8^+^T cells (CD8^+^ GZMH^+^ IL7R^-^ cells) displayed a high expression of the cytotoxic genes GZMH, GZMK, GZMA and NKG7, and low expression of all checkpoint genes and IL7R, which was similar to the T cell subtype previously described by Zheng *et al*^40^; suggesting that these cells are precursors of cytotoxic T cells (online supplemental figure S2C). Cluster 4 (CD8A^+^ HAVCR2^+^ cells) in CD8^+^ T cells highly expressed genes associated with cytotoxicity (GZMA, GZMB, and GZMK) and exhaustion-related markers (TIGHT, HAVCR2, and LAG3), suggesting that these cells are exhausted CD8^+^ T cells (online supplemental figure S2C). Moreover, the total fraction of CD8^+^T subtypes tended to be more abundant in pRCC versus reRCC (19.6% vs 9.4% of total cells, p=0.09, figure 3B). We then validated that the infiltrating level of CD8^+^T cells was significantly higher in pRCC than in reRCC via IHC staining of 41 pairs of pRCC and reRCC samples from the validation cohort (mean:11.02% vs 6.42% of total cells, p=0.005; figure 3C). Furthermore, we found that a high infiltrating level of CD8^+^T cells was significantly associated with longer PFS in RCC patients who received immunotherapy in the SYSUCC immunotherapy cohort (HR 0.45, p=0.033, figure 3D). In the TCGA cohort, higher CD8^+^T cells infiltration was associated with better overall survival (OS) (HR 0.58, p<0.001) and PFS (HR 0.6, p<0.001) in RCC patients (online supplemental figure S2D).

DEGs analysis between pRCC and reRCC indicated that IL2RG was significantly upregulated in CD8^+^T cells in pRCC tissues (figure 3E; online supplemental table S6). Protein γϲ (encoded by IL2RG) was regarded as a component of the receptor for multiple cytokines, including IL-2, IL-4, IL-7, IL-9, IL-15 and IL-21, which could drive T cell proliferation and differentiation.^41^ KEGG pathway analysis showed that CD8^+^T cells in pRCC were characterized by upregulated T cell differentiation, antigen processing and presentation (figure 3F) whereas the upregulated genes of CD8^+^T cells in reRCC tissues were characterized by apoptosis-related genes such as JUN, FOS, FASLG, ACTG1 and CTSC (figure 3E; online supplemental table S6). We also found that the apoptotic pathway was upregulated in CD8^+^T cells of reRCC samples (figure 3G).

In summary, by analyzing the proportion and activated pathways in pRCC and reRCC, we observed a superior anti-tumor immunity of CD8^+^T cells in pRCC than in reRCC tissues.

### Progressive dysfunction of CD8^+^T cells in RCC by trajectory analysis

**Figure 4:**
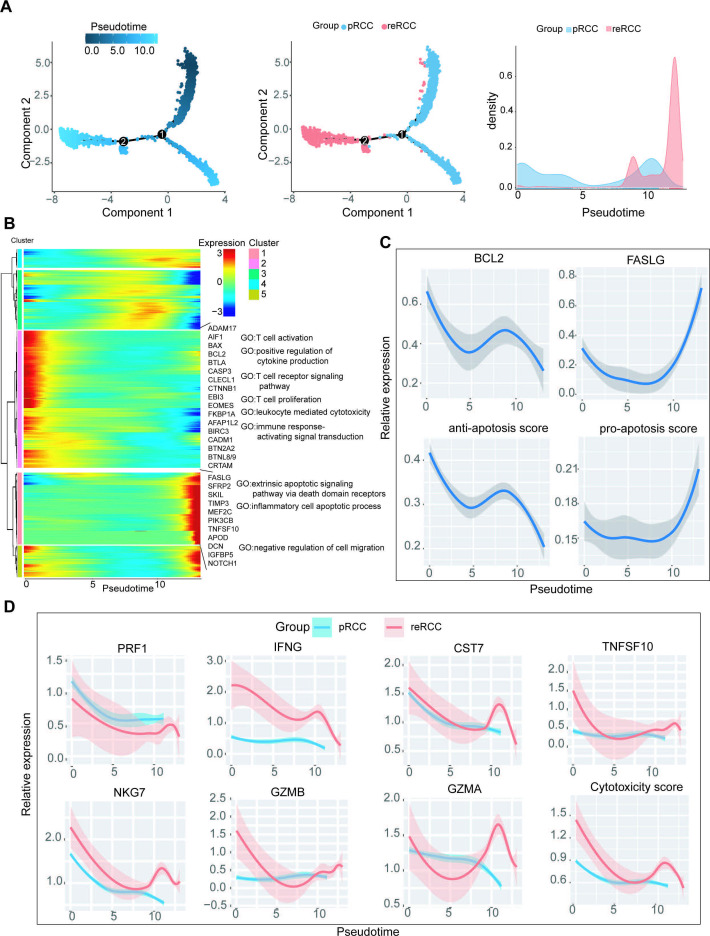
Analysis of CD8 +T cell transition states in pRCC and reRCC samples. (A) Pseudotime-ordered analysis of CD8^+^ T cells from pRCC and reRCC samples. (B) Heatmap showing the dynamic changes in gene expression along the pseudotime. (C) Two-dimensional plots showing the expression scores for genes related to antiapoptosis and proapoptosis along with the pseudotime. (D) Two-dimensional plots showing the dynamic expression of cytotoxicity genes and cytotoxicity score during the CD8^+^ T cell transitions along the pseudotime in pRCC (blue) and reRCC (red) samples. GO, gene ontology; pRCC, primary renal cell carcinoma; reRCC, recurrent renal cell carcinoma.

We explored the dynamic immune states and gene expression of RCC-infiltrated CD8^+^T cells by inferring the state trajectories using Monocle. Most of the CD8^+^T cells were found to be derived from pRCC and showed a high-density peak at both the early and end-stage of pseudotime (figure 4A). We then performed clustering of the genes with pseudotemporal expression pattern, whereby the ordering of genes was clustered into five clusters. Genes in cluster 1, including FASLG, SFRP2 and MEF2C, were highly expressed at the end stage and GO analysis suggested that their function mainly enriched the inflammatory cell apoptotic process, extrinsic apoptotic signaling pathway via death domain receptors, and negative regulation of cell migration. Genes in cluster 2 tended to be downregulated during the pseudotime. GO enrichment analysis showed that their functions were enriched in a series of immune activation functions, such as T cell activation, positive regulation of cytokine production, T cell proliferation, leukocyte mediated cytotoxicity and immune response-activating signal transduction (figure 4B, C).

To further investigate the transition states associated with CD8^+^T cells in reRCC and pRCC samples, we separately analyzed the trajectories of CD8^+^ T cells in pRCC and reRCC samples. CD8^+^T cells in reRCC samples were primarily allocated at the terminal ends in pseudotime trajectories (figure 4A). We next investigated the cytotoxicity changes associated with transitional states. Cytotoxicity-related genes including PRF1, IFNG, CST7, TNFSF10, NKG7, GZMB, and GZMA in CD8^+^ T cell in both pRCC and reRCC samples tended to be downregulated during the pseudotime (figure 4D).

In short, CD8^+^ T cells were progressively dysfunctional with gradually reduced cytotoxicity and increased apoptotic trend, which indicated an immune tolerance status during pseudotime trajectories in pRCC and reRCC.

### CAFs’ expression pattern and clinical outcomes in RCC patients

**Figure 5:**
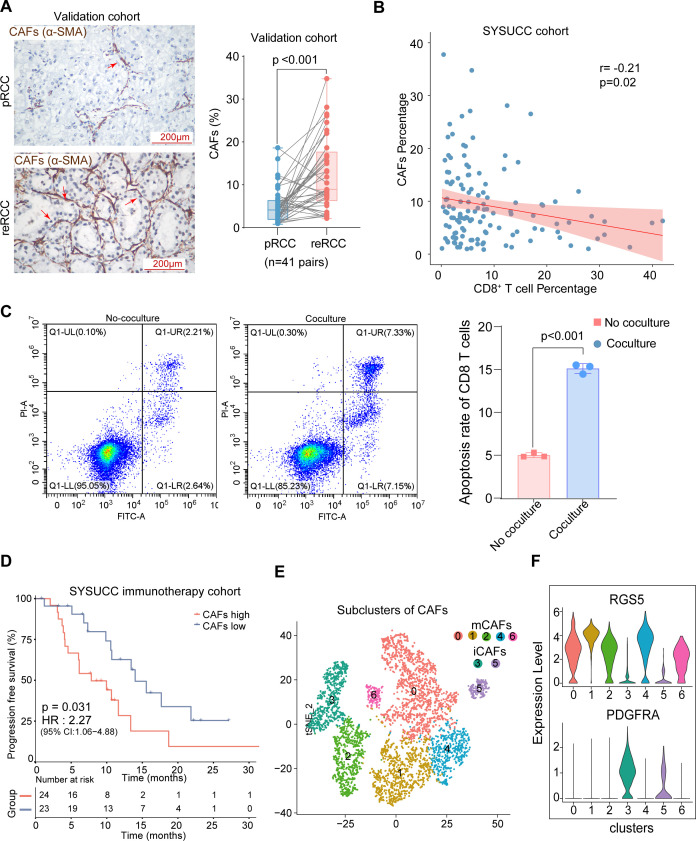
Expression pattern and clinical relevance of CAFs in RCC. (A) IHC staining of α-SMA (a marker of CAFs), in paired pRCC and reRCC samples from the validation cohort. Scale bars, 50 µm. (B) The correlation between IHC staining of α-SMA (a marker of CAFs) and IHC staining of CD8A in 129 RCC patients. (C) Flow cytometric analysis of the proportion of apotostic CD8^+^ T cells, left, CD8^+^ T cells did not coculture with CAFs; right, CD8^+^ T cells cocultured with CAFs. (D) PFS curves according to CAFs (infiltration high or low groups) in tumor tissues in the SYSUCC immunotherapy cohort. The CAFs infiltration high group showed significantly worse PFS. (E) t-SNE projections of subclustered CAFs, labeled in different colors. Cell type annotations are provided in the figure. (F) Violin plots showing the markers of subtypes of CAFs. CAFs, cancer-associated fibroblasts; iCAFs, inflammatory cancer-associated fibroblasts; IHC, immunohistochemistry; mCAFs, Myo-cancer-associated fibroblasts; PFS, progression-free survival; pRCC, primary renal cell carcinoma; reRCC, recurrent renal cell carcinoma; SYSUCC, Sun Yat-sen University Cancer Center; t-SNE, T-distributed stochastic neighbor embedding.

Our scRNA-seq data revealed that compared with pRCC, the infiltrating proportion of CD8^+^ T cells tended decreased and the abundance CAFs tended to be increased in reRCC (figure 1E). By performing IHC staining assay in paired pRCC and reRCC samples, we verified the infiltration of CAFs was significant higher in reRCC samples (12.5% vs 5.2 %, p<0.001, figure 5A). Moreover, we found that CD8^+^ T cells infiltration was negatively correlated with the CAFs infiltrating abundance using IHC staining (r=−0.21, p=0.02) (figure 5B). This led us to hypothesize that the low degree of CD8^+^ T cells infiltration in reRCC could be caused CAFs. We coculture the CAFs and CD8^+^ T cells on Matrigel.^42^ The rate of apoptotic CD8^+^ T cells cocultured with CAFs was increased compared with CD8^+^ T cells culture on Matrigel alone (15.12% vs 5.00 %, p<0.001, figure 5C). We then explored the prognosis significance in RCC patients in SYSUCC immunotherapy cohort and TCGA RCC cohort. A high infiltrating level of CAFs was significantly associated with a poorer PFS in RCC patients who received immunotherapy in the SYSUCC immunotherapy cohort (HR 2.27, p=0.031, figure 5D). Analogously, in the TCGA RCC cohort, higher CAFs infiltration was associated with worse OS (HR 1.49, p=0.011) and PFS (HR 1.44, p=0.007, online supplemental figure S3A).

**Figure 6:**
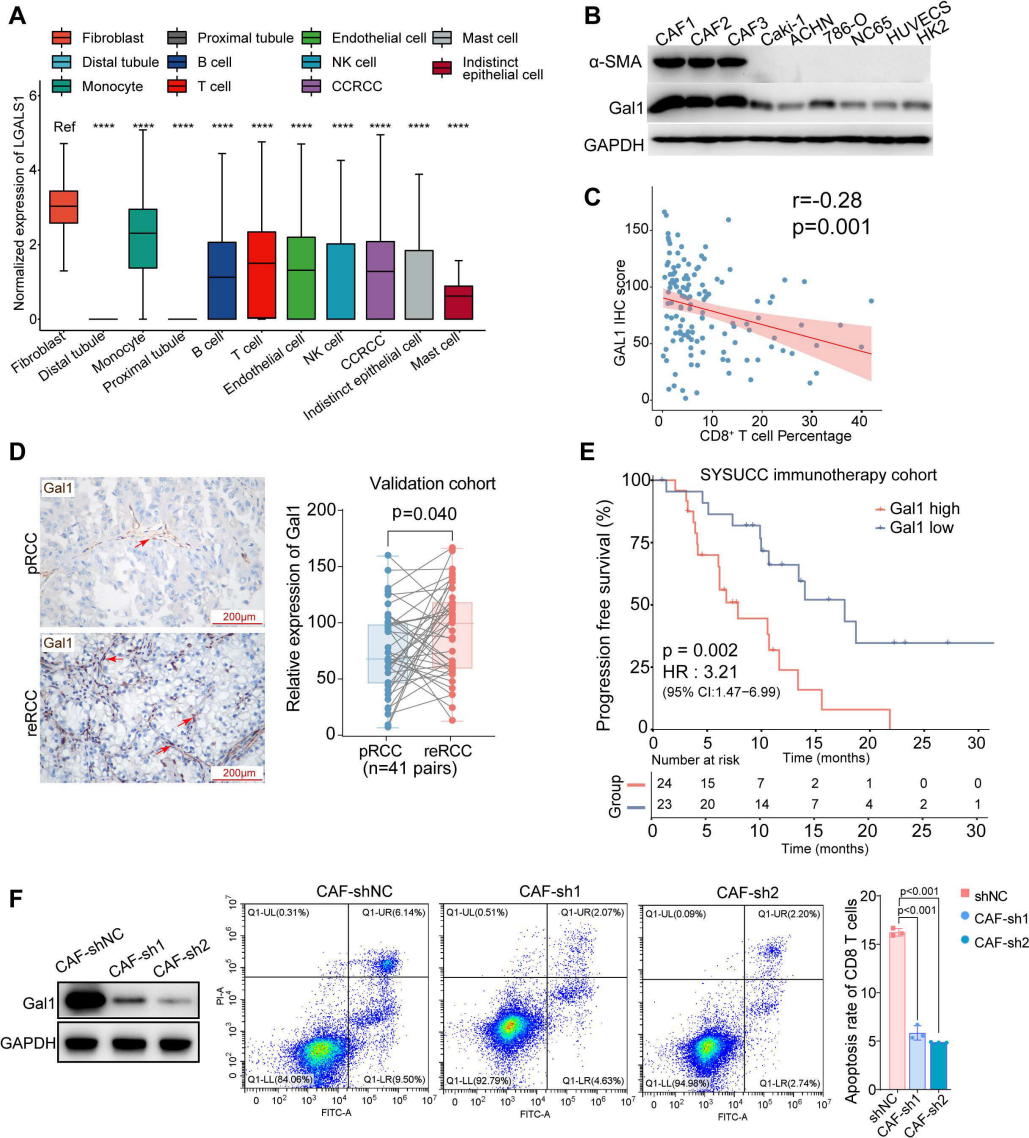
CAFs induced CD8^+^ T cells apoptosis via Gal1. (A) Box plots showing LGALS1 (Gal1) highly expressed in CAFs, compared with other types of cells. (B) Western blotting of α-SMA and Gal-1 in primary CAFs and human RCC cell lines (Caki-1, ACHN, 786-O, and NC65), vein endothelial cell line HUVEC, human kidney cortex/proximal tubule cells (HK2). (C) The correlation between IHC staining of Gal1 and CD8A in 129 RCC patients. (D) IHC staining of Gal1 in paired pRCC and reRCC samples from the validation cohort. Scale bars, 50 µm. (E) PFS curves according to Gal1 (expression high or low groups) in tumor tissues in the SYSUCC immunotherapy cohort. A high expression of Gal1 was significantly associated with a worse PFS. (F) Flow cytometric analysis of the proportion of apotostic CD8^+^ T cells cocultured with control CAFs and Gal1 knock-down CAFs. CAFs, cancer-associated fibroblasts; IHC, immunohistochemistry; PFS, progression-free survival; pRCC, primary renal cell carcinoma; reRCC, recurrent renal cell carcinoma; SYSUCC, Sun Yat-sen University Cancer Center.

To further explore the potential mechanism of promoting tumor progression and immunotherapy resistance of CAFs, we investigated the expression pattern of CAFs. Recluster of CAFs identified seven clusters, including five clusters of myo-CAFs (mCAFs) and two clusters of inflammatory CAFs (iCAFs) according to the expression of RGS5 (mCAFs) and PDGFRA (iCAFs)^43^ (figure 5E, F). Interestingly, we identified significantly increased expression of LGALS1(Gal1) in CAFs (figure 6A), which was widely reported as an immunosuppressive factor that could induce T cell apoptosis.^17 18 21 44^ Then, we noticed that LGALS1 was highly expressed in all seven subtypes of fibroblast (online supplemental figure S3B). To further verify the expression of LGALS1 in CAFs, we isolated and cultured primary CAFs from RCC patients and using multiplexed immunofluorescent staining to confirm a high expression of α-SMA and Gal1(online supplemental figure S3C, D). Next, we confirmed that Gal1 had a higher expression level in CAFs compared with human RCC cell lines (Caki-1, ACHN, 786-O, and NC65), vein endothelial cell line HUVEC, human kidney cortex/proximal tubule cells (HK2) by Western Blotting (figure 6B). We found that lower CD8^+^ T cells infiltration was correlated with higher expression level of Gal1 (figure 6C). Gal1 was expressed higher in reRCC samples than in pRCC samples (figure 6D). For clinical significance, a high expression level of Gal1 was significantly associated with a worse PFS in RCC patients in the SYSUCC immunotherapy cohort (HR 3.21, p=0.002, figure 6E). Further, a high expression level of LGALS1 was associated with a poorer OS (HR 1.71, p<0.001) and PFS in RCC patients in the TCGA cohort (HR 1.88, p<0.001, online supplemental figure S3E).

To investigate that CAFs could trigger CD8^+^ T cells apoptosis via Gal1, we established Gal1 knockdown CAFs (CAF-sh1 and CAF-sh2) cell lines (figure 6F). When CD8^+^ T cells cocultured with Gal1 knockdown CAFs (CAF-sh1 and CAF-sh2) on Matrigel, the rate of apoptotic CD8^+^ T cells was decreased, compared with CD8^+^ T cells cocultured with control CAFs (5.85% vs 16.08%, 5.41% vs 16.08%, p<0.001, figure 6F).

We thereby concluded that immunosuppressor Gal1 was substantially expressed in CAFs. Upregulation of Gal1, as well as abundant CAFs, predicted a poor prognosis in RCC patients receiving immunotherapy. And CAFs could trigger CD8^+^ T cells apoptosis by Gal1.

### CAFs promote tumor growth and hamper immunotherapy efficacy by expressing Gal1 in vivo

**Figure 7:**
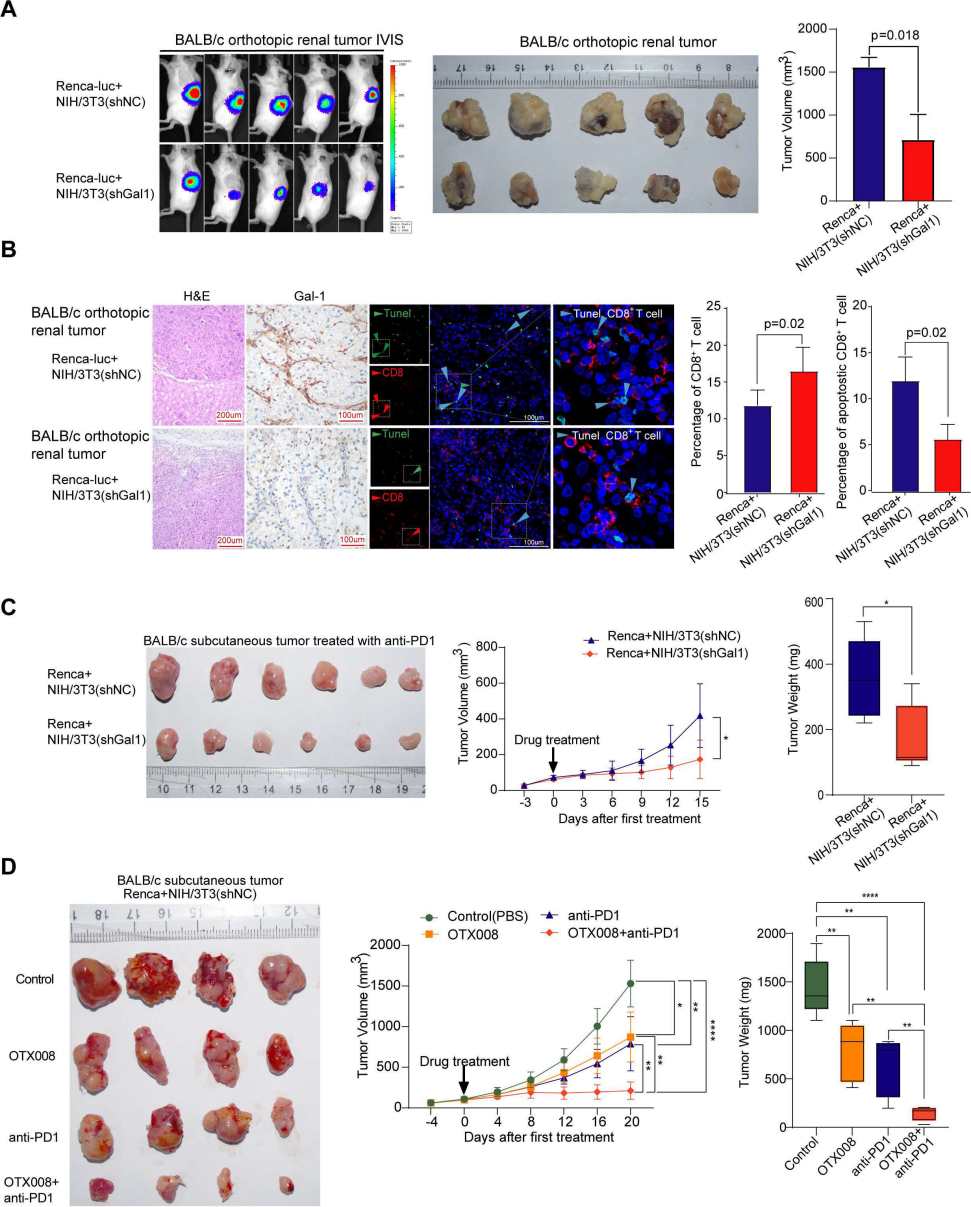
Gal1 inhibition reduces CD8^+^ T cells apoptosis and enhances immunotherapy efficacy in RCC model. (A) IVIS imaging of orthotopic renal tumors of Renca-luc cells cocultured with Gal1 -knocked-down NIH/3T3 (shGal1) fibroblasts, or Renca-luc cells cocultured with negative control NIH/3T3 (shNC) fibroblasts in BALB/c mice. Barplot showing the volume of tumors (right). (B) H&E staining (left). IHC staining of Gal1(middle), multiple immunofluorescence staining of CD8A and TUNEL(right) in orthotopic renal tumors from BALB/c mice. Barplot showing the percentage of infiltrating CD8^+^ T cells and the ratio of apoptotic CD8^+^ T cells to total CD8^+^ T cells. (C) Tumor growth curve for Renca cells plus NIH/3T3 (shNC) or Renca cells plus NIH/3T3 (shGal1) tumors treated with anti-PD1 antibody (n=6 mice/group). (D) Tumor growth curve for Renca cells plus NIH/3T3 (shNC) treated with PBS, OTX008, anti-PD1 antibody, and OTX008 combined anti-PD1 antibody (n=4 mice/group). Data are presented as the mean±SD. *P<0.05, **p<0.01, ****p<0.0001. IHC, immunohistochemistry; IVIS, bioluminescent imaging system; PBS, phosphate-buffered saline.

To further evaluate the relationship between Gal1 expressed in CAFs and immunosuppression, in vivo experiments were performed in immune-competent BALB/c mice. We knocked down Gal1 in NIH/3T3 cell line, as shRNA2 had better efficiency in knocking down Gal1 expression (online supplemental figure S3F), we labeled NIH/3T3 cell transduced with shRNA2 as NIH/3T3(shGal1). Luciferase-expressing mouse-derived cell line (Renca-luc) and NIH/3T3(shGal1) were used for these experiments. We established two groups: cancer cells (Renca -luc) plus NIH/3T3 (shNC) as the control group, and cancer cells plus with Gal1 knocked down NIH/3T3 (Renca-luc +NIH/3T3(shGal1)) as the research group. Cancer cells and fibroblasts were inoculated into the left kidney cortex of each mouse, and tumor growth was assessed using a bioluminescent imaging system (IVIS). Orthotopic tumors were harvested after 4 weeks, and tumor volume analysis revealed that tumors were significantly smaller when Gal1 level was knocked down in NIH/3T3 cells in BALB/c mice (p=0.018, figure 7A). These results suggested that CAFs could promote tumor growth by expressing Gal1. To further assess whether knocking down Gal1 could influence the CD8^+^ T cell infiltration and apoptosis, we tested the abundance and apoptosis of infiltrating CD8^+^ T cells in the orthotopic tumors of the two groups in BALB/c mice by multiplexed immunofluorescence staining for CD8A and TUNEL. In mice implanted with Renca-luc plus NIH/3T3-shGal1, the infiltrating level of CD8^+^ T cells was significantly higher (17.62% vs 12.59%, p=0.02, figure 7B) and the ratio of apoptotic CD8^+^ T cells was significantly decreased (5.76% vs 12.12%, p=0.02, figure 7B), compared with the group implanted with Renca-luc plus NIH/3T3(shNC). These in vivo data further supported that CAFs could promote tumor growth and induce CD8^+^ T cell apoptosis by expressing Gal1.

Next, we investigated whether Gal1 expression level in CAFs could impact the antitumor effect of anti-PD1 in vivo. Renca +NIH/3T3(shGal1) or Renca +NIH/3T3(shNC) cells were subcutaneously injected into the right flank of BALB/c mice. Our findings showed that tumor volume and weight were significantly lower when Gal1 was knocked down in NIH/3T3 (p<0.05, figure 7C). These results showed that knocking down Gal1 in NIH/3T3 substantially improved the tumor growth inhibition effect of anti-PD1 therapy in vivo.

### Gal1 Specific inhibitor (OTX008) combined with anti-PD-1 enhanced the efficacy of immunotherapy in vivo

BALB/c mice subjected to subcutaneous injection of Renca +NIH/3T3(shNC) cells were randomly divided into four groups: (1) control, (2) OTX008 alone, (3) anti-PD1 alone, and (4) combined OTX008 and anti-PD1. The results showed a significant reduction in tumor growth compared with the control when administered with OTX008 alone (p<0.05), anti-PD1 alone (p<0.001), and the combined treatment (p<0.001). Interestingly, the combination of OTX008 and anti-PD1 exerted the most potent effect in inhibiting tumor growth. (figure 7D).

## Discussion

Recurrent tumors are often treated based on the molecular and pathological features of the primary tumor, especially in patients who have lost the opportunity for surgical resection or inability to obtain pathological specimens. However, studies comparing primary versus recurrent tumors suggested that secondary tumors can be extremely different from the primary tumor.^34 45^ Sun *et al* described a distinctly different TME between primary and relapse hepatocellular carcinoma using scRNA-seq.^26^ However, the difference in expression pattern and TME between pRCC and reRCC remained undeciphered. Given the high heterogeneity and the poor prognosis of advanced RCC patients, including reRCC, it is necessary to investigate the difference between pRCC and reRCC as precisely as possible.

In this study, we observed that reRCC was characterized by abundant CAFs and low numbers of CD8^+^ T cells infiltration in the TME using scRNA seq data and validated the results by IHC staining in 41 paired pRCC and reRCC samples. There was a negative correlation between CD8^+^ T cells and CAFs infiltration abundance. Remarkably, we identified that LGALS1 (Gal1), a well-known immunosuppressor,^17 18^ was highly expressed in CAFs in RCC, especially in reRCC. And Gal1 expression in RCC was significantly correlated with CD8^+^ T cells infiltration. By coculture experiments, we indicated that CAFs could trigger CD8^+^ T cells apoptosis by Gal1. Clinically, we found that high infiltrating level of CD8^+^ T cells was associated with better prognosis, whereas upregulation of Gal1, as well as abundant CAFs, was associated with poor PFS in RCC patients who received immunotherapy. In vivo, knocking down Gal1 in CAFs could suppress tumor growth, increase CD8^+^ T cells infiltration, reduce the proportion of apoptotic CD8^+^ T cells, and enhance the efficacy of immunotherapy. We elucidated that the combinational treatment of anti-PD1 with OTX008, the specific inhibitor of Gal1,^46^ could improve immunotherapy efficacy compared with those administered with anti-PD1 alone.

Our data showed reduced CD8^+^ T cells infiltration in the TME of reRCC. This indicated that reRCC could be characterized as a cold tumor, which is defined by a low degree of T cell and CD8^+^ T cell infiltration and the downregulation of immune checkpoints such as PD-1, PD-L1, and LAG3(48). CD8^+^ T cells play a central role in anti-tumor immunity, and clinical evidence has proved that cold tumor was poorly responsive to immunotherapy.^11^ Whereas immunotherapy could be more effective in hot tumors, which are characterized as having high CD8^+^ T cell density.^15 47 48^ In renal clear cell carcinoma, baseline CD8 +T cell infiltration does not predict response to immunotherapy,^49 50^ however, the lack of an association between PFS in RCC patients received immunotherapy and the presence of CD8^+^ cells should not be interpreted to indicate that these elements are irrelevant but more likely indicate that other factors are also contributing to biological responses to immunotherapy.^50^ In the phase 3 JAVELIN Renal 101 trial,^50^ although no association between PFS and CD8^+^ cells detected by IHC in tumor region was observed in the combination arm (avelumab plus axitinib), their gene expression deconvolution results indicated that CD8^+^ T cells had a significant interaction term with PFS in patients received avelumab plus axitinib, which further reinforce the clinical significance of cytotoxic T cells in advanced RCC. In our study, increased CD8^+^ T cell infiltration was associated with longer PFS (HR 0.45, p=0.033), compared with patients with lower CD8^+^ T cell infiltration. Similar results were found in our analysis of the TCGA-KIRC cohort.

Meanwhile, we found a high level of CAFs infiltration in reRCC. CAFs is an important contributor of ECM structure and have multiple ways to promote tumor progression.^16^ CAFs could remodel the immunosuppressive tumor-infiltrating lymphocytes population of the TME by secreting a high level of IL-6 in esophageal cancer.^15^ Interestingly, IL-6 secreted by CAFs could significantly enhance the malignancy of intrahepatic cholangiocarcinoma cells by promoting stem-like properties.^13^ However, in our data, IL-6 had extremely low expression in CAFs as well as in other cell types in RCC (online supplemental figure S3G), which indicated the heterogeneity of CAFs among tumor types. In RCC, CAFs were highly prevalent cells in the TME and were involved in facilitating tumor cell proliferation, angiogenesis, metastasis, and therapy resistance.^51 52^ Studies have shown that CAFs infiltrating the TME could hamper the response of immunotherapy in metastatic bladder, melanoma, and kidney cancer.^12 53^ Based on the analysis of 47 patients who received immunotherapy in this study, significantly worse PFS was found in patients with high CAFs infiltration, compared with patients with low CAFs infiltration. Thus, our study provided additional evidence on immunotherapy resistance mediated by CAFs.

To further explore the potential mechanism of CAFs immunosuppression, the expression pattern of CAFs was investigated. Interestingly, we found that LGALS1 had highest expression level in CAFs among cell types in RCC. LGALS1 encodes the secretory protein Gal1, which is confirmed to promote tumor progression through a variety of mechanisms, such as angiogenesis,^54^ epithelial-mesenchymal transformation,^24^ and immunosuppression.^19 23^ In 1995, Perillo *et al*^17^ first reported that Gal1 induced apoptosis of activated human T cells in vitro. Our in vivo study showed that the ratio of apoptotic CD8^+^ cells was significantly reduced and the CD8^+^ T cells infiltration was increased in the tumor model implanted with Renca plus NIH/3T3(shGal1). However, a study conducted by Nambiar *et al*^23^ indicated that Gal1 secreted by endothelium remodeled immunosuppression TME via T cell exclusion rather than apoptosis, and other reports showed inconsistencies in the Gal1 apoptotic effect depending on the tumor model.^44 55^ And it was reported that stromal cell in ECM is able to directly kill susceptible T cells via Gal1.^42^ In the coculture experiments in our study, when CD8^+^ T cells and CAFs cocultured together directly on Matrigel, the rate of apoptotic CD8^+^ T cells was increased and could be inhibited by Gal1 knockdown, which indicated that CD8^+^ T cell death was Gal1 dependent.

Immunotherapy has brought hope to patients with advanced RCC while the majority of patients still do not have durable responses to these agents,^7–9 33^ and efficacy has been compromised in cases where CD8^+^ T cells infiltration is low.^15 47 48^ In such cases, combination therapy strategies could be envisaged to both increase the CD8^+^ T cells infiltration and stimulate preexisting immune cell function. We found that knocking down Gal1 in CAFs could alter the TME from a cold tumor to a hot tumor, and enhance the efficacy of immunotherapy in vivo. These results implied the rationality of combination immunotherapy with targeting Gal1 in tumors with abundant infiltrating CAFs or overexpression of Gal1. In our study, we proved that OTX008 and anti-PD1 combination therapy could inhibit tumor growth in murine renal cancer models, compared with those treated with anti-PD1 alone. Furthermore, the specific inhibitor of Gal1, OTX008, has completed the first phase of its clinical trial (https://ichgcp.net/clinical-trials-registry/NCT01724320), which could facilitate the conduction of further clinical trials of OTX008 on RCC. Taking together, these findings provided novel insights into the combination of immunotherapy with other drugs. Gal1 expression showed the potential to be a predictor for the efficacy of immunotherapy in RCC, and targeting Gal1 combined with immunotherapy could improve the prognosis of patients with RCC.

Despite the important findings observed in this study, there were some limitations to be clarified. First, the scRNA-seq data of primary tumor and adjacent normal kidney tissues was downloaded from public datasets, although we strictly referenced the methods of the original literature mentioned when preparing our own single-cell suspension of recurrent tumor tissues and minimized the batch effects during analysis. Second, the pRCC and reRCC samples for scRNA-seq were limited and unpaired, although we validated the results in 41 paired pRCC and reRCC samples, the results of scRNA-seq data need to be further validated. Third, we demonstrated a progressively dysfunctional state of CD8^+^ T cells with gradually reduced cytotoxicity in RCC through trajectory analysis. However, given the importance of post-transcriptional regulatory mechanisms, the mRNA expression level may not provide a true reflection of the protein expression in CD8^+^ T cells.^11^ Finally, although our results could bring new insights into the combination of immunotherapy with OTX008, the efficacy of such regimens are yet to be validated in clinical trials.

### Conclusion

Our study delineated the heterogeneity between pRCC and reRCC, described a low CD8^+^ T cell and high CAFs infiltration TME in reRCC, and highlighted CAFs as a suppressor of CD8^+^ T cell via expressing Gal1. Thus, targeting Gal1 could enhance immunotherapy response, and combinational treatment of anti-PD1 with OTX008 could improve immunotherapy efficacy. Our work provides a novel mechanism of CAFs in mediating an inhibitory immune microenvironment and sheds light on a unique approach to improve the results of immunotherapy in RCC.

## Data Availability

Data are available on reasonable request. All the data used in this study could be available from the author Yulu Peng (E-mail: pengyl1@sysucc.org.cn).
