# Supplementary material for: Single-cell transcriptomics reveals a low CD8+ T cell infiltrating state mediated by fibroblasts in recurrent renal cell carcinoma
Source: J Immunother Cancer. 2022 Feb 4;10(2):e004206. doi: 10.1136/jitc-2021-004206 (PMC8819783; doi:10.1136/jitc-2021-004206)
Supplement: Supplementary data [file jitc-2021-004206supp002.pdf]

Single-cell transcriptomics reveals a low T cell infiltrating state mediated by fibroblasts in recurrent renal carcinoma

Methods

Human subject

Two tumor tissues from patients diagnosed with local recurrent clear cell renal carcinoma (reRCC) in Sun Yat-Sen University Cancer Center (SYSUCC) were used for scRNA-seq. Three perioperatively primary renal cell carcinoma (pRCC) tissues were obtained during nephrectomy and used for isolation and culture of CAFs. Formalin-fixed paraffin-embedded (FFPE) tissue blocks of pairwise primary and recurrent RCC were collected from 41 patients in Sun Yat-Sen University Cancer Center(SYSUCC, n=21, Guangzhou, China), Peking University Third Hospital (n=11, Beijing, China), Shanghai Jiao Tong University affiliated Renji Hospital (n=3, Shanghai, China), The First Affiliated Hospital of Sun Yat-Sen University (n=2, Guangzhou, China), The Third Xiangya Hospital of Central South University (n=2, Changsha, China), Nanfang Hospital, Southern Medical University(n=2, Guangzhou, China) as the validation cohort. 47 RCC patients who received immunotherapy in our center were included in the SYSUCC immunotherapy cohort. We defined progression-free survival (PFS) as no progression/relapse after immunotherapy use. The available clinical features of the SYSUCC immunotherapy cohort are summarized in **supplementary table S1**.

DEGs and pathways analysis

Differentially expressed gene analysis between pRCC and reRCC was conducted using "FindMarkers" function, implemented in the Seurat package, with log-scaled fold change > 0.5 and p value < 0.05 (Wilcoxon Rank Sum test). Pathways of DEGs in malignant cells were explored using HALLMARK gene sets and KEGG gene sets were utilized in pathways enrichment of DEGs in $CD8^+$ T cells.

Samples collection and initial processing

reRCC tissues were obtained from two patients diagnosed with local relapsed clear cell renal cell carcinoma in SYSUCC. Samples were directly obtained from the operating room during surgery. We strictly referenced the methods described by Young et al.[1] when preparing our own single-cell suspension. Tissues were rinsed in HBSS (Gibco) until there was no visible blood then sliced into approximately 30 mm^3 pieces of tissue and digested for 30 min at 37°C with agitation in a digestion solution containing 25μg/mL Liberase TM (Roche) and 50μg/ml DNase (Sigma) in RPMI (Gibco). Sample dissociation solutions were filtered by a 70-μm cell strainer, washed with PBS, and live cells enriched using a Dead Cell Removal Kit (Miltenyi Biotec) as per manufactures instructions. The single-cell suspension was stained with 0.4% trypan blue (Thermo Fisher Scientific) to examine the concentration of live cells. Cell suspensions immediately proceed to single-cell library preparation.

Library preparation and sequencing

Cellular suspensions were loaded on a 10X Genomics GemCode Single-cell instrument that generates single-cell Gel Bead-In-EMlusion (GEMs). Libraries were generated and sequenced from the cDNAs with Chromium Next GEM Single Cell 3’Reagent Kits v3.1. Single-cell transcriptomic amplification and library preparation were performed by Genedenovo Biotechnology Co., Ltd (Guangzhou, China) using single-cell 3′ v3 (10X Genomics). The libraries were then pooled and sequenced across six lanes on an Illumina NovaSeq 6000 system (Illumina, Inc., San Diego, California, US). Pre-processing of scRNA-seq fastq files was conducted using Cell Ranger v3.0.2 (10X genomics). scRNA-seq reads were aligned to the hg19 reference genome (ref-version 3.0.0), and the count matrix of cell barcodes by genes used for downstream analysis was generated using the Cell Ranger count function with parameter–expect-cells=3000.

Public data Acquisition

The scRNA-seq count matrix of 2 pRCC and 3 adjacent normal kidney tissues described by Young et al.[1] were downloaded and used, as this is the first study characterizing pRCC using scRNA-seq. For the TCGA cohort, the expression matrix, along with the clinical information of the patients containing 539 tumor samples and 72 normal samples were downloaded from The Cancer Genome Atlas (TCGA, https://portal.gdc.cancer.gov/) Kidney Renal Clear Cell Carcinoma (KIRC) dataset. The EPIC package (1.1.5) [2] was used to calculate the infiltration abundance of $CD8^+$ T cells and CAFs in the TCGA cohort.

Single-cell gene expression quantification and subcluster detection.

Raw gene expression matrices were imported and processed using the Seurat R package (version 3.1.5) [3]. Low-quality cells were removed following 3 measurements: 1) cells had either fewer than 200 or over 6000 unique molecular identifiers (UMIs), over 20,000 or less than 200 expressed genes or over 15% UMIs derived from the mitochondrial genome, or over 2.5% UMIs derived from the erythrocytic genome; 2) cells had an average expression level of less than 2 for a curated list of housekeeping genes[4]; 3) cells had a co-expression of EPCAM and PTPRC. 4) Doublets were detected by DoubletFinder R package (version 2.0.3) [5]for single sample and manually detected the doublets in re-clustering the cell types. 7 seurat objects containing 2 reRCC and 2 pRCC and 3 normal samples were then merged, and batch effects were minimized using Harmony R package (version 1.0)[6]. Gene expression matrices of the remaining high-quality cells were normalized to the total cellular UMI counts. The normalized expression was scaled (scale. factor = 10,000) by regressing out the total cellular UMI counts and percentage of mitochondrial gene. Highly variable genes (top 2000) were extracted using the Seurat FindVariableGenes function. Then, we performed principal component analysis (PCA) analysis using high variable genes, and significant PCs (top 20) were selected to perform dimension reduction. Clusters were found using FindClusters function (dims.use = 1:20, resolution = 0.5). The tSNE analysis was used for dimension

reduction and visualization of gene expression[7].

CNV analysis

Initial CNVs were estimated by the expression levels of genes within each chromosome region using the inferCNV R package[8]. The CNVs of epithelial cells were calculated by expression level from scRNA-seq data for each cell with a cutoff of 0.1. We further employed K-Means clustering in all epithelial cells to distinguish malignant and non-malignant epithelial cells based on CNV scores. The CNV score of each cell was calculated as quadratic means of (CNV region -1). Then, we distinguished malignant depended on the class of K-Means and cells distribution in groups.

Pseudotime trajectory analysis

The cell lineage trajectory of $CD8^{+}$T was inferred using the Monocle2 R package[9]. We used the “differentialGeneTest” function to derive DEGs from each cluster and genes with a q-value < 0.01 were used to order the cells in pseudotime analysis. After the cell trajectories were constructed, we then identified the genes that varied according to pseudotime using the “differentialGeneTest” function in Monocle2 and used them to perform GO enrichment analysis. We used the average expression (measured by log2 (TPM + 1) of anti-apoptosis, pro- apoptosis related genes to define the anti-apoptosis and pro-apoptosis score for $CD8^{+}$ T cells. The anti-apoptosis and pro-apoptosis related genes were listed in **Supplementary Table S2**.

Isolation and culture of CAFs from RCC tissues.

Briefly, primary RCC tissues were obtained from the operating room during nephrectomy. Tissues were rinsed in HBSS (Gibco) until there was no visible blood then sliced into approximately 30 mm^3 pieces of tissue and digested for 30 min at 37°C with agitation in a digestion solution containing 25μg/ml Liberase TM (Roche) and 50μg/ml DNase (Sigma) in RPMI (Gibco). Sample dissociation solutions were

filtered by a 40-μm cell strainer and were neutralized with complete medium and centrifuged at 300g for 5 min. The cell pellet was suspended in Dulbecco's Modified Eagle Medium (DMEM) containing 10% selected fetal bovine serum (FBS) and 1 ng/ml basic fibroblast growth factor (bFGF, R&D Systems, Minneapolis, MN), 100 μg/ml penicillin, and 100 U/ml streptomycin (Invitrogen, NY, USA). The cells were grown in the culture dishes. After 48h, non-adherent cells were removed and washed twice with phosphate-buffered saline (PBS). Adherent fibroblasts were further incubated for 5-8 days until 80-90% confluence. Then, the mixed fibroblasts were labeled by α-SMA (Sigma, Cat: A2547, 1:100) and sorted by FACS to enrich CAFs. The CAFs were further expanded in the above medium (DMEM +10% FBS) and passage 5~10 CAFs were used in this study.

IHC analysis

After deparaffinization, slides were hydrated in alcohol and endogenous peroxidase activity was quenched for 30 min in 10% hydrogen peroxide. Antigen epitope retrieval was induced by microwave heating. To examine the expression pattern of candidate antibodies in RCC tissues, sections were immunostained with primary antibodies for 1 hour at 37°C. Then, enzyme-labeled goat anti-mouse/rabbit IgG polymer (Cat: PV-6000, ZSGB-BIO, Beijing) was added and incubated at room temperature for 20 minutes. The signal was detected using DAB (ZSGB-BIO, Beijing), following the protocol of the manufacturer. The counts of CAFs, $CD8^+$ T cells and the IHC score of Gal1was calculated by HALO software (Indicalab, New Mexico, USA). The antibodies were listed as follows: CD8A (Abcam, ab237710, 1:1000); α-SMA (Sigma, Cat: A2547, 1:800); (Gal1, NSJ bioreagents, Cat: R31653, 1:1000)

Western Blotting.

Western blotting was performed as previously described [7]. Briefly, the cells were lysed in RIPA buffer (50 mM Tris-HCl, pH 7.5, 150mM NaCl, 1% NP-40, 0.5%

sodium deoxycholate, 0.1% SDS) and protease cocktail inhibitor I (Calbiochem, San Diego, CA). The cell lysates were separated on SDS-PAGE (10% or 12.5% polyacrylamide, EpiZyme, Shanghai) and transferred to polyvinylidene difluoride membranes (PVDF, Roche). The blots were blocked in TBST containing 5% skimmed milk and incubated with primary antibodies overnight at 4˚C. The membranes were washed with TBST 4×10 minutes and then incubated with secondary antibodies (Beyotime, Shanghai, China) for 1 h at room temperature. After three additional washes with TBST, immunoreactive bands were detected using enhanced chemiluminescence (Tanon, Shanghai, China), followed by exposure on ChemiDoc Touch Imaging System (BIO-RAD, Hercules, CA). The antibodies were listed as follows: CD8A (Abcam, ab237710, 1:1500); α-SMA (Sigma, Cat: A2547, 1:1000); (Gal1, NSJ bioreagents, Cat: R31653, 1:1000)

Immunofluorescence co-staining of CD8A and TUNEL in murine tumors.

Following excision from mice, tumor tissues were washed by PBS and fixed in 4 % paraformaldehyde overnight at room temperature, then embedded in the paraffin by standard procedures. Tissue blocks were sectioned at 4-μm thickness on a microtome, placed onto positively charged glass slides, and stained with H&E according to standard protocols. For immunofluorescence co-staining of CD8A, PANO 4-plex IHC kit (Panovue, 0079100020) was used. CD8A (Abcam, ab217344, 1:800) primary antibodies was incubated overnight at 4˚C, followed by horseradish peroxidase-conjugated secondary antibodies incubation and tyramide signal amplification. The slides were microwave heat-treated after tyramide signal amplification operation. Nuclei were stained with 4-6-diamidino-2-phenylindole (DAPI). Then incubated with TUNEL (Beyotime, C1086) for an hour at 37 ˚C. Images were captured by a confocal laser microscope (OLYMPUS FV1000, Tokyo, Japan), and counts of $CD8^+$ T cells and apoptotic $CD8^+$ T cells were calculated by HALO software (Indicalab, New Mexico, USA).

Mice and cell lines

Female BALB/c mice were purchased from Vital River (Beijing, China) and were maintained in specific pathogen-free conditions in the animal laboratory of SYSUCC. The murine cell line of renal cancer (Renca) was kindly provided by Professor Luo (Center for Precision Medicine, Sun Yat-sen University, Guangzhou, China), and the murine fibroblast line (NIH/3T3) was purchased from the National Collection of Authenticated Cell Cultures of China. Renal cancer-associated fibroblasts were isolated from human primary clear cell renal carcinoma as described previously.

Establishment of stable Gal1 knocking fibroblasts

Lentiviral vectors containing luciferase, Gal1 small hairpin (sh) RNA plasmid were purchased from Genechem Co., LTD (Shanghai, China) and prepared in accordance with standard protocols. Briefly, 3×10^6 293T cells were seeded in a 10 cm dish. After 24 hours incubation, cells were transfected with shRNA or negative control plasmids by Lipofectamine 3000 (Invitrogen). We changed the medium to remove transfection reagents after 8 hours and virus was collected 36 hours later. NIH/3T3 cells and human CAFs were transduced with sh-Gal1 or sh-NC lentiviruses for 24 hours. After 48 hours of lentivirus infection, cells were screened using puromycin (1 microg/ml) for 7 days to establish stably transduced cell lines. Cell lysates were prepared for protein extraction and then cell line with lower Gal1 protein expression was chosen (based on the Western blotting results) for further experiments.The shRNA target sequences for murine Gal1were: sh1: GTGTGTAACACCAAGGAAGAT and sh2:AGACGGACATGAATTCAAGTT. And The shRNA target sequences for human Gal1in were: sh1: GCCCACGGCGACGCCAACACC and sh2: GCTGCCAGATGGATACGAATT.

Isolation of human CD8$^+$ T cells

Peripheral blood mononuclear cells (PBMC) from health donor were first isolated from buffy coat by density gradient centrifugation using Lymphocyte Separation Medium (Biosharp). CD8$^+$ T cells were then isolated from PBMC using CD8

microbeads (Miltenyi Biotec), and purity was assessed by flow cytometry. Subsequent experiments were carried out after confirming $CD8^+$ T cell population was >95% pure. For all the experiments, $CD8^+$ T cells were cultivated with X-VIVO medium (LONZA) supplemented with 10% fetal bovine serum (FBS) at the density of 1.5×10^6 cells per ml per cm^2 with additional interleukin-2 supplement (100 IU/mL, Miltenyi Biotec.) and ImmunoCult™ Human CD3/CD28 T Cell Activator (10μl/mL, InvivoGen).

CAFs and $CD8^+$ T cells coculture system and flow cytometry analysis

It was reported that stromal cell in extracellular matrix is able to directly kill susceptible T cells via Gal1[10]. To investigate whether CAFs could induce $CD8^+$ T cells apoptosis and its potential regulators. We designed CAFs and CD8+ T cells coculture system according to previous study[10]. 5×10^5 CAFs in 2 ml of media were plated directly on solidified Matrigel (400 μl) in 6-well plates for 24 h, 3×10^6 $CD8^+$ T cells were added to Matrigel solidified and allowed to bind the Matrigel for 6 h. Media with unbound cells was aspirated and collected for analysis, Matrigel was digested by incubation in 800 μl of dispase (Invitrogen) for 40 min at 37°C. Digested cells incubated in 1 ml 2.5% Trypsin for 2 min at 37°C, and passed over a 40-μm filter. Digested cells and unbound cells were washed twice with PBS. $CD8^+$ T cells were then isolated using CD8 microbeads (Miltenyi Biotec) and then incubated with FITC Annexin V and PI at room temperature in the dark for 15 minutes. $CD8^+$ T cells apoptosis was assessed by flow cytometry.

***In vivo* experiments**

To evaluate CAFs decreased the infiltrated $CD8^+$ T cells in the tumor via expressing Gal1, we used 4 - 6 weeks old female BALB/c mice. Mice were anesthetized and 100μL of a single-cell suspension containing either Renca-luc (1×10^6 cells) with NIH/3T3 (Gal1-shNC) fibroblasts (1×10^6 cells), or Renca-luc (1×10^6 cells) with NIH/3T3 (shGal1) fibroblasts (1×10^6 cells) was injected into the right kidney cortex,

the renal orthotopic tumor were detected by luciferin (VivoGlo Luciferin, In Vivo Grade). Mice were sacrificed after 4 weeks by cervical dislocation under anesthesia, and tumors were harvested, and tumor volume were examined.

To analyze whether Gal1 expressed in CAFs could hamper the efficacy of immunotherapy *in vivo*, Renca (1×10^6 cells) with NIH/3T3 (shNC) fibroblasts (1×10^6 cells), or Renca (1×10^6 cells) with NIH/3T3 (shGal1) fibroblasts (1×10^6 cells) resuspended in 100 µl PBS were injected into the skin of the right flank of 4–6 weeks old male BALB/c mice[11]. Tumor growth was monitored every 3 days by measuring the length and width of the tumor. After tumors reaching approximately 50 mm^3, anti-PD1 (clone RMP1-14, BioXCell) was injected i.p. (200µg/mouse) every 2 days[12].

To investigate the Gal1 inhibitor OTX008 could increase the efficacy of immunotherapy, BALB/c mice were injected subcutaneously in the right flank with Renca (1×10^6 cells) and NIH/3T3 (shNC) fibroblasts (1×10^6 cells), when tumors reached 50 mm^3, mice were randomly assigned to 4 groups, namely control (vehicle), OTX008 (Selleck, 10mg/kg/mouse/2 days, administered intraperitoneally)[13], anti-PD1 (200µg/mouse /2 days, administered intraperitoneally) and OTX008 + anti-PD1.

The tumor volume was calculated using the formula: $0.52 \times \text{Length} \times \text{Width}^2$.

Statistical analysis

The data were analyzed using GraphPad Prism software version 8 (GraphPad Software, San Diego, California, US). Comparisons between paired pRCC and reRCC were performed with the paired Wilcoxon test. Others were performed with unpaired Wilcoxon test. We constructed survival curves using the Kaplan-Meier method, and their equality was compared via log-rank test. The tumor growth in mice was measured using the method of a repeated-measures ANOVA. All data are presented as means ± standard deviations (SDs). * $p < 0.05$, ** $p < 0.01$, *** $p < 0.001$. Statistical significance was set at p values less than 0·05.

Study approval

This study was performed in accordance with the ethical standards of the Helsinki Declaration and the ethical guidelines for Medical and Health Research Involving Human Subjects[14]. Written informed consent was obtained for each of the participant patients. All cases were de-identified and personal identifiable details were removed from their case descriptions to ensure anonymity. This study was approved and reviewed by the Ethics Review Board of Sun Yat-sen University Cancer Center. Mouse experiments were performed in a specific pathogen-free environment at the animal laboratory of the SYSUCC according to institutional guidelines, and all animal experimental protocols were approved and reviewed by the Ethics Review Committee for Animal Experimentation of SYSUCC. All experiments were performed in accordance with the guidelines and regulations indicated by these committees.

Refrence

[1] Young MD, Mitchell TJ, Vieira Braga FA, Tran MGB, Stewart BJ, Ferdinand JR, Collord G, Botting RA, Popescu DM, Loudon KW, Vento-Tormo R, Stephenson E, Cagan A, Farndon SJ, Del Castillo Velasco-Herrera M, Guzzo C, Richoz N, Mamanova L, Aho T, Armitage JN, Riddick ACP, Mushtaq I, Farrell S, Rampling D, Nicholson J, Filby A, Burge J, Lisgo S, Maxwell PH, Lindsay S, Warren AY, Stewart GD, Sebire N, Coleman N, Haniffa M, Teichmann SA, Clatworthy M and Behjati S. Single-cell transcriptomes from human kidneys reveal the cellular identity of renal tumors. Science 2018; 361: 594-599.

[2] Racle J, de Jonge K, Baumgaertner P, Speiser DE and Gfeller D. Simultaneous enumeration of cancer and immune cell types from bulk tumor gene expression data. Elife 2017; 6:

[3] Stuart T, Butler A, Hoffman P, Hafemeister C, Papalexi E, Mauck WM, 3rd, Hao Y, Stoeckius M, Smibert P and Satija R. Comprehensive Integration of Single-Cell Data. Cell 2019; 177: 1888-1902 e1821.

[4] Zhang M, Yang H, Wan L, Wang Z, Wang H, Ge C, Liu Y, Hao Y, Zhang D, Shi G, Gong Y, Ni Y, Wang C, Zhang Y, Xi J, Wang S, Shi L, Zhang L, Yue W, Pei X, Liu B and Yan X. Single-cell transcriptomic architecture and intercellular crosstalk of human intrahepatic cholangiocarcinoma. J Hepatol 2020; 73: 1118-1130.

[5] McGinnis CS, Murrow LM and Gartner ZJ. DoubletFinder: Doublet Detection in Single-Cell RNA Sequencing Data Using Artificial Nearest Neighbors. Cell Syst 2019; 8: 329-337 e324.

[6] Korsunsky I, Millard N, Fan J, Slowikowski K, Zhang F, Wei K, Baglaenko Y, Brenner M, Loh PR and Raychaudhuri S. Fast, sensitive and accurate integration of single-cell data with Harmony. Nat Methods 2019; 16: 1289-1296.

[7] Tirosh I, Izar B, Prakadan SM, Wadsworth MH, 2nd, Treacy D, Trombetta JJ, Rotem A, Rodman C, Lian C, Murphy G, Fallahi-Sichani M, Dutton-Regester K, Lin JR, Cohen O, Shah P, Lu D, Genshaft AS, Hughes TK, Ziegler CG, Kazer SW, Gaillard A, Kolb KE, Villani AC, Johannessen CM, Andreev AY, Van Allen EM, Bertagnolli M, Sorger PK, Sullivan RJ, Flaherty KT, Frederick DT, Jane-Valbuena J, Yoon CH, Rozenblatt-Rosen O, Shalek AK, Regev A and Garraway LA. Dissecting the multicellular ecosystem of metastatic melanoma by single-cell RNA-seq. Science 2016; 352: 189-196.

[8] Patel AP, Tirosh I, Trombetta JJ, Shalek AK, Gillespie SM, Wakimoto H, Cahill DP, Nahed BV, Curry WT, Martuza RL, Louis DN, Rozenblatt-Rosen O, Suva ML, Regev A and Bernstein BE. Single-cell RNA-seq highlights intratumoral heterogeneity in primary glioblastoma. Science 2014; 344: 1396-1401.

[9] Qiu X, Mao Q, Tang Y, Wang L, Chawla R, Pliner HA and Trapnell C. Reversed graph embedding resolves complex single-cell trajectories. Nat Methods 2017; 14: 979-982.

[10] He J and Baum LG. Presentation of galectin-1 by extracellular matrix triggers T cell death. J Biol Chem 2004; 279: 4705-4712.

[11] Kato T, Noma K, Ohara T, Kashima H, Katsura Y, Sato H, Komoto S, Katsube R, Ninomiya T, Tazawa H, Shirakawa Y and Fujiwara T. Cancer-Associated Fibroblasts Affect Intratumoral CD8(+) and FoxP3(+) T Cells Via IL6 in the Tumor Microenvironment. Clin Cancer Res 2018; 24: 4820-4833.

[12] Moynihan KD, Opel CF, Szeto GL, Tzeng A, Zhu EF, Engreitz JM, Williams RT, Rakhra K, Zhang MH, Rothschilds AM, Kumari S, Kelly RL, Kwan BH, Abraham W, Hu K, Mehta NK, Kauke MJ, Suh H, Cochran JR, Lauffenburger DA, Wittrup KD and Irvine DJ. Eradication of large established tumors in mice by combination immunotherapy that engages innate and adaptive immune responses. Nat Med 2016; 22: 1402-1410.

[13] Koonce NA, Griffin RJ and Dings RPM. Galectin-1 Inhibitor OTX008 Induces Tumor Vessel Normalization and Tumor Growth Inhibition in Human Head and Neck Squamous Cell Carcinoma Models. Int J Mol Sci 2017; 18:

[14] Ogasawara K. [8. Revised "Ethical Guidelines for Medical and Health Research Involving Human Subjects"].
