# Supplementary material for: Single-cell transcriptomics reveals a low CD8+ T cell infiltrating state mediated by fibroblasts in recurrent renal cell carcinoma
Source: J Immunother Cancer. 2022 Feb 4;10(2):e004206. doi: 10.1136/jitc-2021-004206 (PMC8819783; doi:10.1136/jitc-2021-004206)
Supplement: Supplementary data [file jitc-2021-004206supp003.pdf]

**Supplementary Figures and figure legends.**

## Figure S1

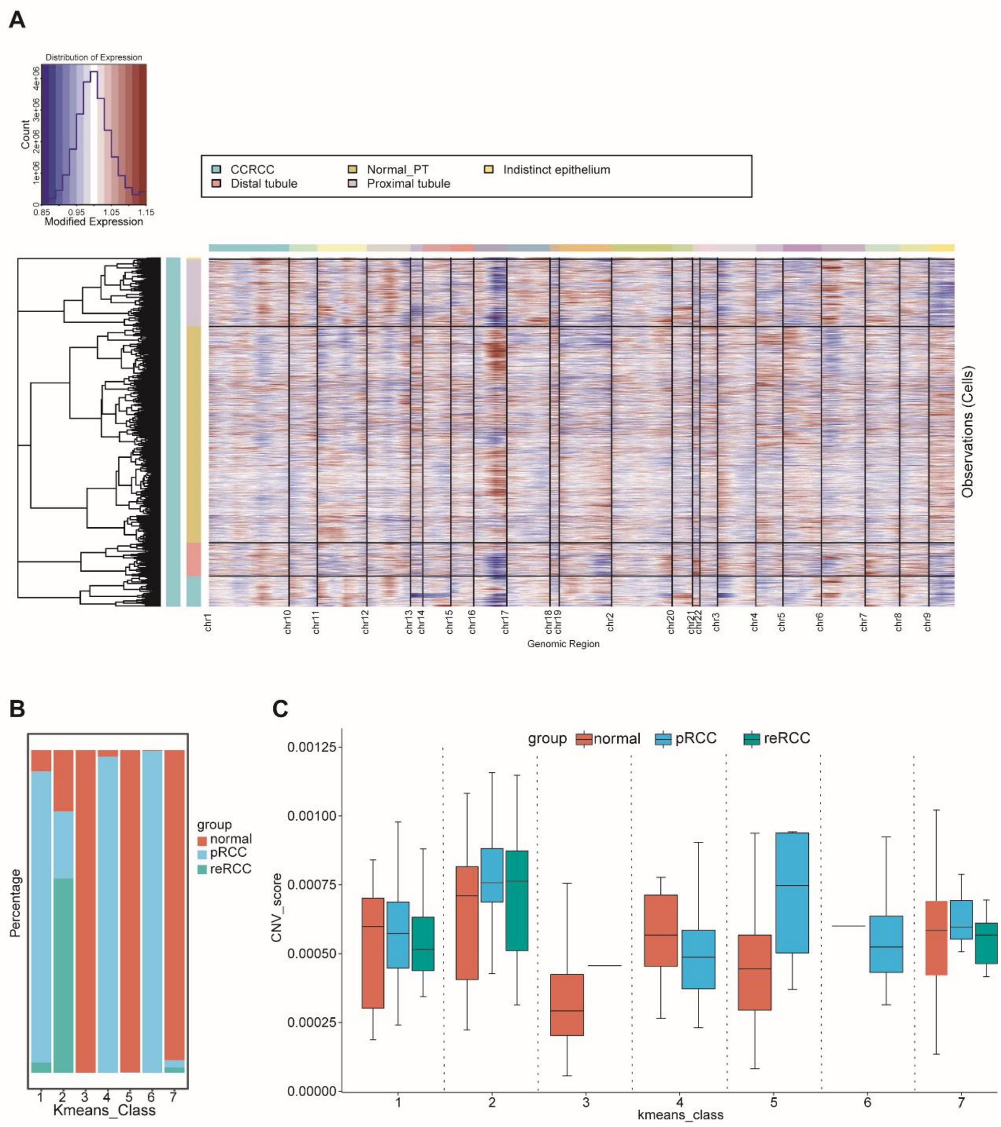


**Figure S1 Detecting malignant cells using CNV analysis and kmeans clustering.**

A. CNV analysis of epithelial cells.

B. Histogram showing the percentage of cell in kmeans class by groups.

C. Box plots showing the CNV score for kmeans class in each group.

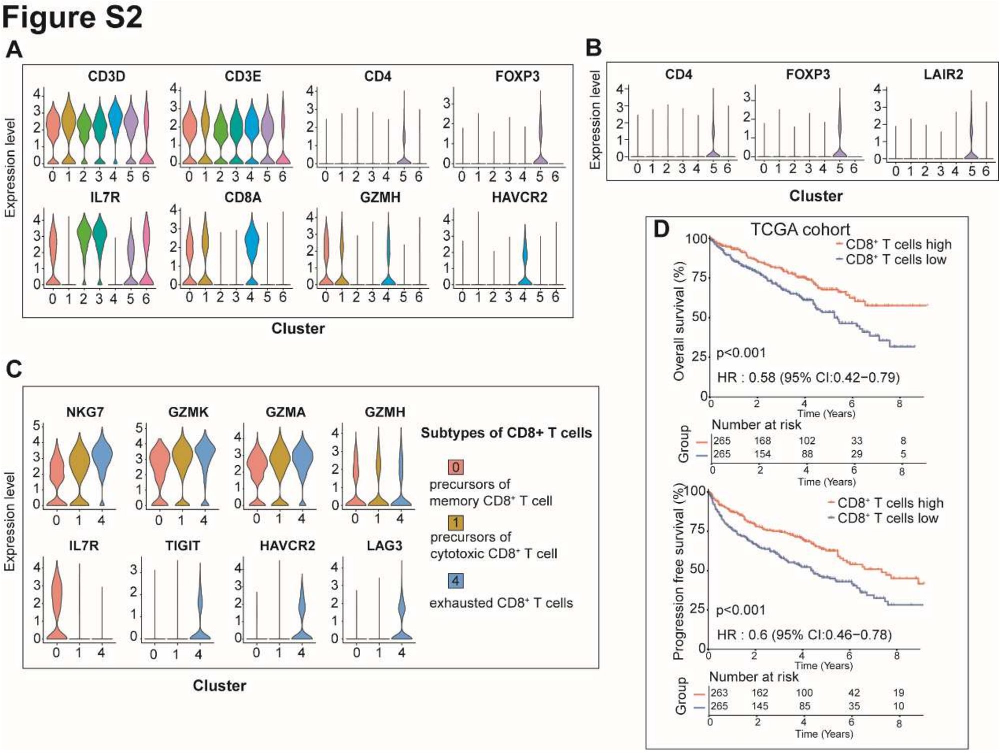


**Figure S2**

A. Violin plots showing the markers of subtypes of T cells.

B. Violin plots showing the markers of Tregs were specifically expressed in cluster5 of T cells.

C. Violin plots showing the genes of cytotoxicity were specifically expressed in $CD8^{+}$ T cells, and marker genes of 3 subtypes of $CD8^{+}$ T cells.

D. K-M curves showed a high infiltrating level of $CD8^{+}$T cells was significantly associated with longer overall survival and progression-free survival in RCC patients in the TCGA cohort.

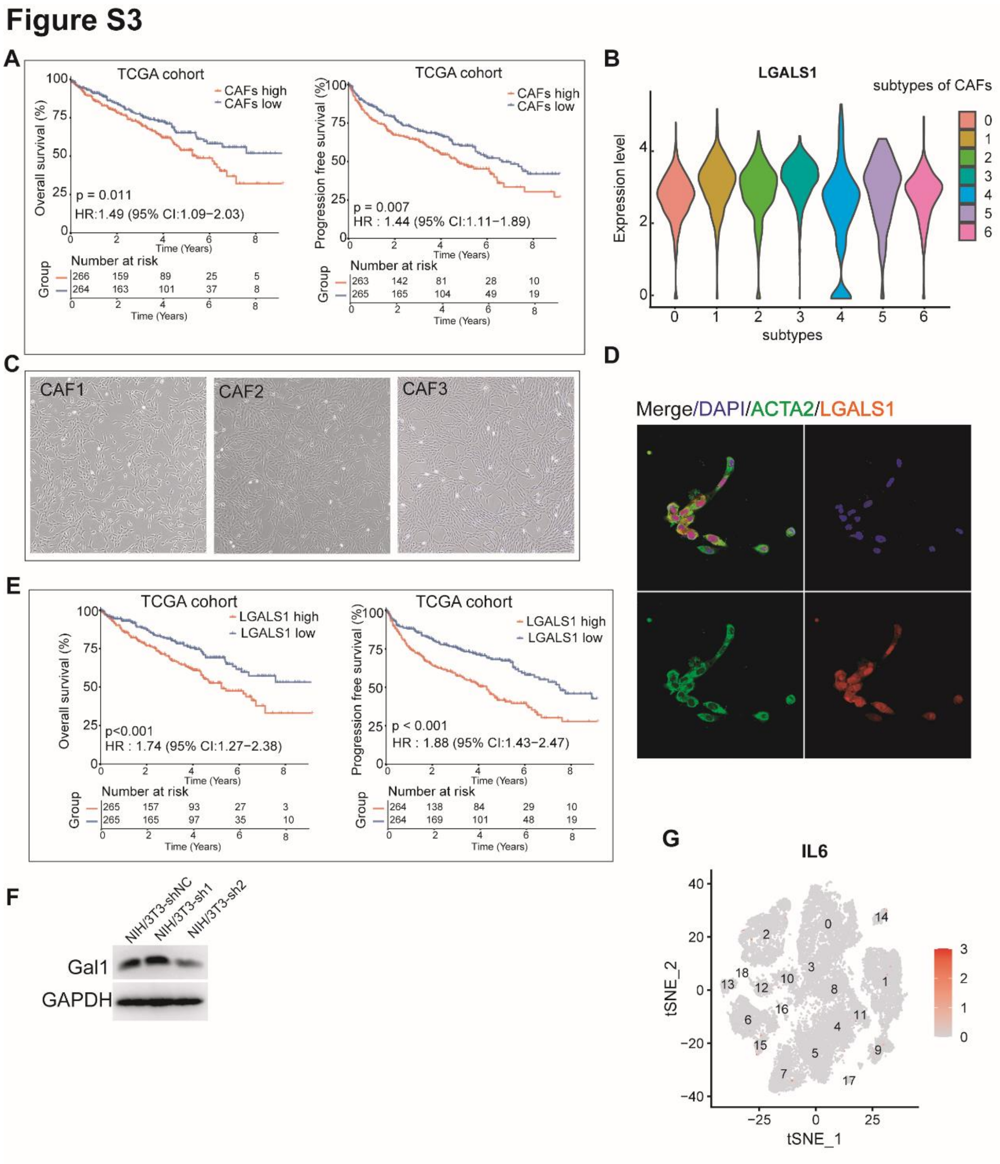


**Figure S3**

A. K-M curves showed a high infiltrating level of CAFs was significantly associated with poorer overall survival and progression-free survival in RCC patients in the TCGA cohort.

B. LGALS1 expressed high in 7 subclusters of CAFs.

C. Isolating and culturing primary CAFs from RCC patients

D. Multiplexed immunofluorescent staining of α-SMA and Gal-1 in primary CAFs.

E. K-M curves showed a high expression level of Gal-1 was significantly associated with poorer overall survival and progression-free survival in RCC patients in the

TCGA cohort.

F. Establishing and validating knocking down Gal1 in NIH/3T3 cell lines.

G. tSNE plot showing IL6 expressed extremely low in all cell types in RCC.
